# Supplementary material for: Nanopore blockade sensors for ultrasensitive detection of proteins in complex biological samples
Source: Nat Commun. 2019 May 8;10:2109. doi: 10.1038/s41467-019-10147-7 (PMC6506515; doi:10.1038/s41467-019-10147-7)
Supplement: Supplementary file 1 — Supplementary Information [file 41467_2019_10147_MOESM1_ESM.pdf]

Supplementary Information for

Nanopore blockade sensors for ultrasensitive detection of proteins in complex biological samples

Chuah & Wu et al.

Supplementary Note 1: Fabrication of the solid-state nanopores

Supplementary Figure 1: Fabrication and chemical modification of solid-state nanopores and magnetic nanoparticles.

Supplementary Note 2: Characterisation of the solid-state nanopores

Supplementary Figure 2: Nanopore diameter can be controlled by varying the electron beam exposure dose during EBL step.

Supplementary Figure 3: Solid-state nanopore profile analysis.

Supplementary Note 3: Ionic current through solid-state nanopores

Supplementary Figure 4: Ionic current through solid-state nanopores.

Supplementary Figure 5: Noise analysis of solid-state nanopores.

Supplementary Note 4: Chemical modification of solid-state nanopores

Supplementary Figure 6: XPS characterisation of chemically modified SiN surfaces.

Supplementary Table 1: Prepared surfaces used in antifouling and DSC coupling studies.

Supplementary Figure 7: Ionic current analysis of unmodified and silane-EG_6 modified solid-state nanopores.

Supplementary Figure 8: Ionic current noise analysis of unmodified and silane-EG_6 SAM modified solid-state nanopores.

Supplementary Figure 9: Continuous ionic current trance of a 27 nm nanopore in 100 mM KCl and 10 mM Tris pH 7.4 when a potential bias of 100 mV is applied.

Supplementary Note 5: Estimate of the number of anti-PSA antibody sites on a single MNP as well as a single nanopore

Supplementary Note 6: Characterisation of the magnetic nanoparticles

Supplementary Figure 10: Iron oxide magnetic nanoparticles.

Supplementary Figure 11: SEM image of a MNP sitting inside a SiN nanopore.

Supplementary Note 7: Finite Element Analysis

Supplementary Figure 12: Finite element analysis of electric potential and field distributions inside a nanopore.

Supplementary Figure 13: Electric potential and field distributions inside a nanopore.

Supplementary References

Supplementary Note 1: Fabrication of the solid-state nanopores

The method used to fabricate the solid-state nanopores.

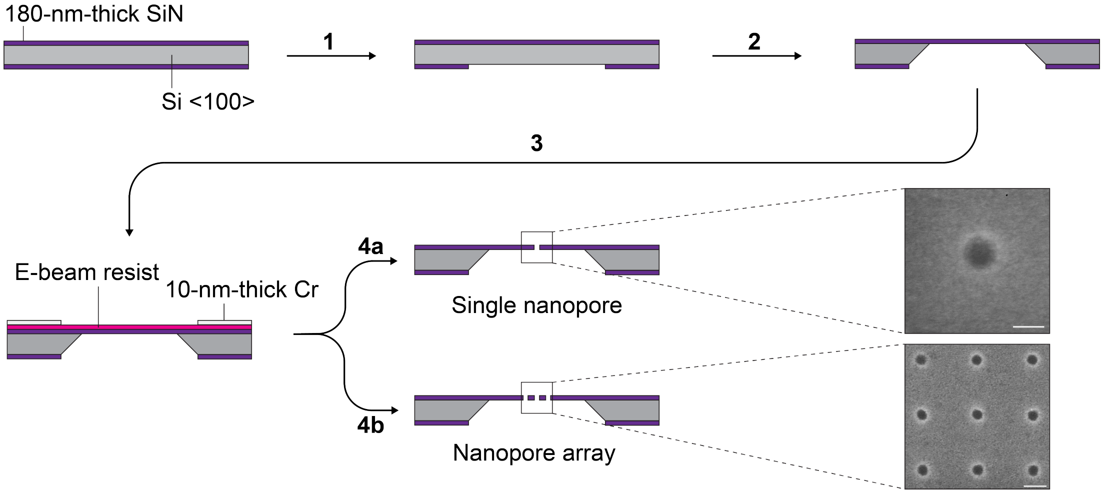

Supplementary Figure 1. Fabrication and chemical modification of solid-state nanopores and magnetic nanoparticles. Schematic of the steps used for fabrication of solid-state nanopores in silicon nitride (SiN) membranes using photolithography, electron beam lithography (EBL) and reactive ion etching (RIE): **1**. Photolithography and CF_4 reactive ion etching. **2**. KOH chemical etching. **3**. Spin coat e-beam resist and thermal evaporation of Cr. **4a**. Single e-beam dot exposure, **4b**. multiple e-beam dot exposure, and subsequently CF_4 reactive ion etching. Insets: scanning electron micrographs of nanopores fabricated in SiN membrane: (top) a 48 nm nanopore, scale bar: 100 nm; (bottom) a 3 × 3 array of nanopores with 0.5 μm spacing between the pores, scale bar: 200 nm.

Supplementary Note 2: Characterisation of the solid-state nanopores

The relationship between nanopore diameter and electron beam exposure dose is elucidated in Supplementary Fig. 2 it reveals an inverse exponential decay dependency. These results demonstrate that EBL is very suitable for fabricating nanopores with diameters >20 nm with high reproducibility and direct control over the size of the nanopores.

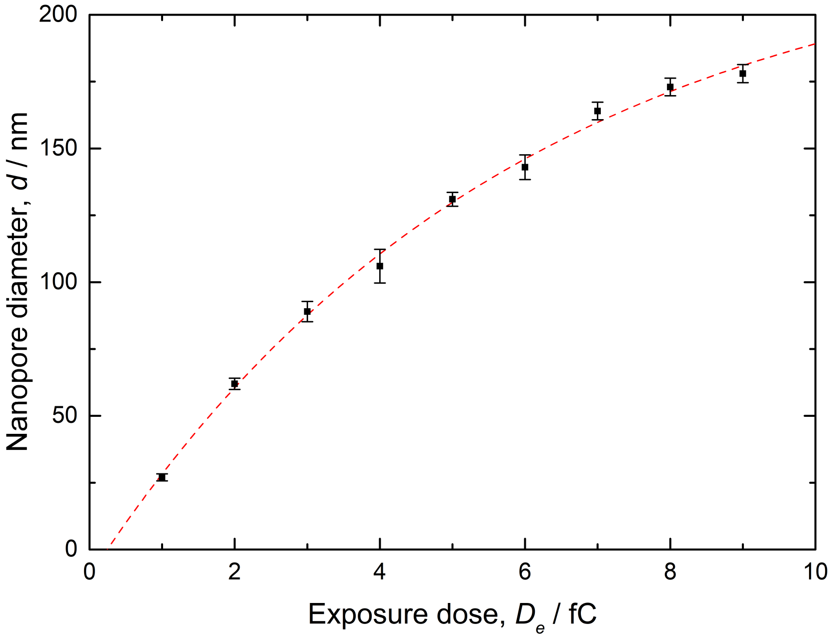

Supplementary Figure 2. Nanopore diameter can be controlled by varying the electron beam exposure dose during EBL step. Plot of the measured nanopore diameter under SEM, d, as a function of the electron beam exposure dose used, D_e. Dashed red line represents a fit of $d \propto D_e^{\gamma}$, where γ = 0.84. The error bars are defined as the standard deviation between measurements.

Taking SEM images and plotting the grey values of the cross-sectional intensity profile as a function of distance from the centre of a pore, one can further elucidate the internal geometry of the nanopores. Supplementary Figure 3 shows examples of two nanopores 55 nm and 62 nm in diameter with their corresponding intensity profiles shown in Supplementary Fig. 3(c) and (d). As from the previous inference, the bright ring surrounding a nanopore corresponds to a sloped surface in the nanopore while the decreasing contrast gradient towards the centre of the pore reaches a minimum grey value that represents the pore void. Since the thickness of SiN membrane layer is known, the slope angle of the pore wall can be calculated from the ratio of the diameters of the pore measured at the top (bright ring region) and bottom (lowest grey value) of the SiN membrane using basic trigonometry as depicted in Supplementary Fig. 3(e) and (f). After examining 10 nanopore samples of various diameters fabricated from different batches, it was found that the nanopore profile resembles a truncated cone structure

with an estimated sidewall angle of 70° ± 2° (mean ± s.d., n=10). This is equivalent to a full cone with a 40° aperture from its tip. Wei *et al.* also attained a similar truncated conical pore profile from their solid-state nanopores fabricated with EBL and RIE by reconstructing the three dimensional structure of a nanopore using electron tomography[1]. They obtained a sidewall angle of 71°, which consistent with the angle measured experimentally in this work.

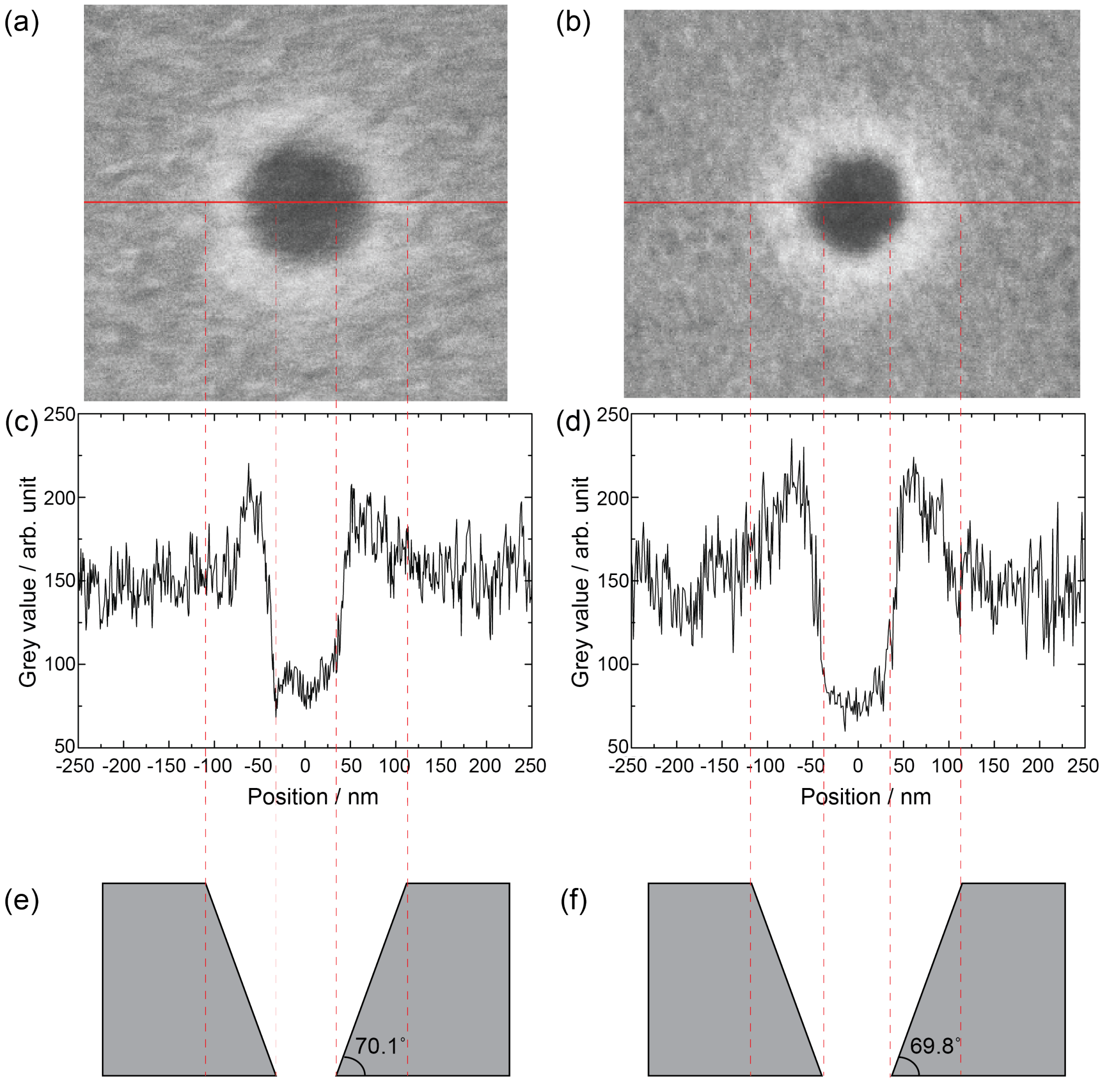

Supplementary Figure 3. Solid-state nanopore profile analysis. SEM images of solid-state nanopores with diameters (**a**) 55 nm and (**b**) 62 nm. Solid red lines represent the cross-sectional signal intensity profiles analysed. (**c** – **d**) Plots of grey value of the intensity profiles as a function of distance from the center of nanopores. (**e** – **f**) Cross-sectional illustration of the deduced nanopore profile based on intensity profile analysis. Dashed red lines are visual

guides aiding correlation between the SEM images, intensity profile plots and nanopore profile diagrams.

Supplementary Note 3: Ionic current through solid-state nanopores

In multiple nanopore systems, Gadaleta *et al.* observed that the total ionic conductance for the pores is dependent on the number of pores present in the membrane and that the averaged ionic conductance per pore strongly decreases with increasing number of pores[2]. Deviation from the linear scaling of ionic conductance is not ideal for the proposed nanoparticle-based nanopore sensor as this would compromise the read-out mechanism of system, whereas a nanopore blocked by a nanoparticle is expected to produce a predictable characteristic and discrete drop in the measured ionic current. Sub-linear dependency on the ionic conductance to the number of pores was not observed in the 2 × 2 and 3 × 3 nanopore arrays shown in Supplementary Fig. 3.

This is attributed to the 3 μm spacing between adjacent pores in the nanopore arrays being sufficiently large that minimal overlap of access resistance regions occurs. That is for two dimensional nanopore arrays, if the number of pores, N, is approximately greater than the ratio of the distance between adjacent pores, L, multiplied by pore length, l and pore diameter, d, squared, the access resistance effect will dominate over bulk conductance and thus the sub-linear scaling of ionic conductance will take effect[2].

$$N \approx (L\frac{l}{d^2})^2 \quad (1)$$

In this case, N was found to be about 550,000 where L = 3 μm, l = 180 nm and d = 27 nm. As a result, the ionic conductance of each individual pore in the array tends to resemble that of isolated pores.

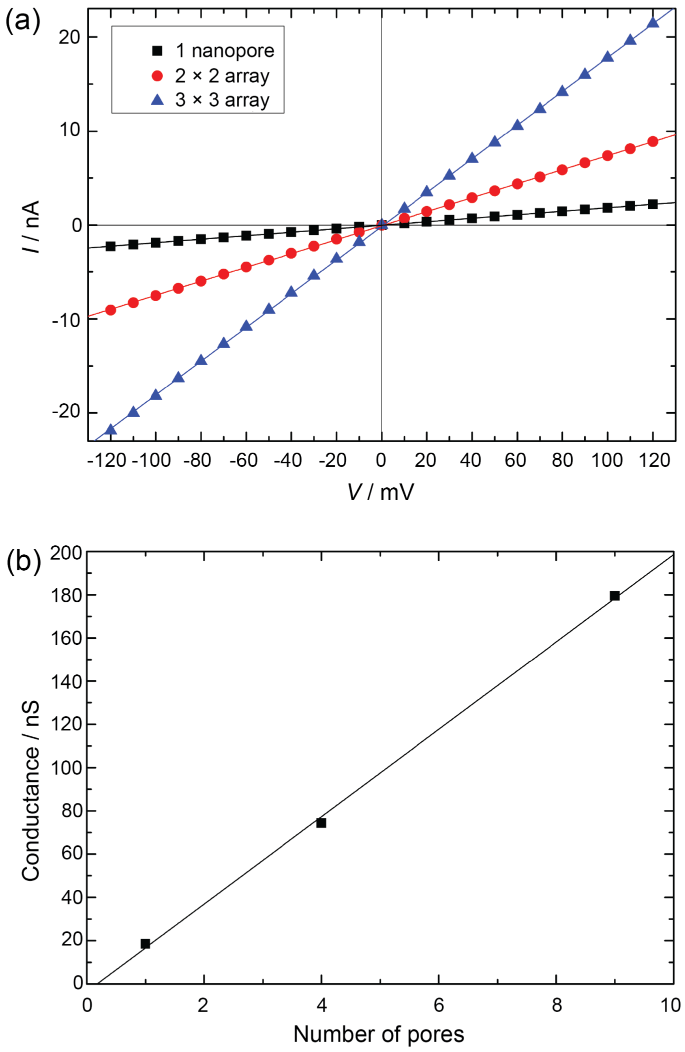

Supplementary Figure 4. Ionic current through solid-state nanopores. (**a**) *I*-*V* curves of a 27 nm nanopore, 2 × 2 array and 3 × 3 array of 27 nm nanopores in SiN membrane. For the nanopore arrays, the pores are spaced 3 μm apart from each other. (**b**) A plot of ionic conductance of the nanopore(s) versus the number of pores present in the SiN membrane. Solid lines are linearly fitted to the measured data points. All ionic currents were measured in 100 mM KCl and 10 mM Tris pH 7.4.

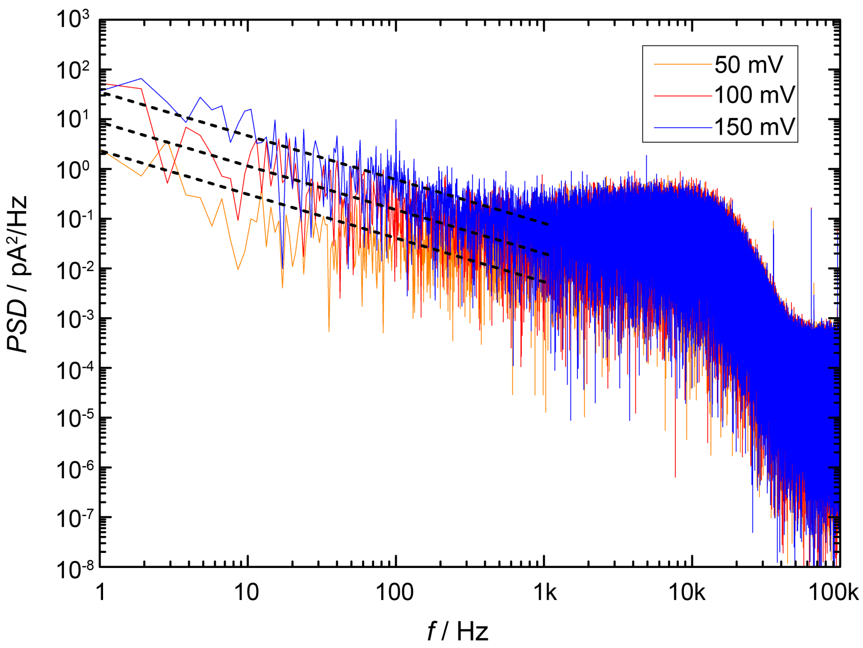

Supplementary Figure 5. Noise analysis of solid-state nanopores. Power spectrum density (PSD) of a 27 nm SiN nanopore at an applied potential bias of 50 mV (orange), 100 mV (red) and 150 mV (blue) in 100 mM KCl and 10 mM Tris pH 7.4. Black dashed lines are fitted to $S^2(f) \propto 1/f$.

Supplementary Note 4: Chemical modification of solid-state nanopores

Supplementary Figure 6 compares piranha-treated SiN surface to silane-EG_6 SAM modified SiN surfaces before and after activation by DSC (the schemes for these surfaces present in Supplementary Table 1). In Supplementary Fig. 6(a), residual peaks in the C region was detected on an unmodified SiN surface due to presence of carbonaceous contaminants on the surface while Supplementary Fig. 5(b) shows a peak in the N 1s at 398 eV characteristic to SiN. Following modification of the SiN surface with an OH-terminated silane-EG_6 SAM, the emergence of two strong peak signals at 284.9 eV and 286.7 eV was observed in Supplementary Fig. 6(c). The former peak corresponds to the etheric C typical of EG units while the latter peak indicates the presence of the propylic chain that separates the EG_6 chain from the silane group. Therefore, these data confirmed the silane-EG_6 SAM was indeed grafted onto the SiN surface.

After activation of hydroxyl terminal group of the silane-EG_6 SAM by DSC, the appearance of two C 1s peaks at higher binding energies in Supplementary Fig. 6(e) as well as a N 1s signal at 401 eV in Supplementary Fig. 6(f) which were not present previously can be attributed to the presence of succinimidyl carbonate groups. The ratio of the area under the peaks at 288.8 eV due to the two carbons from C=O of the imide groups and the peak at 287.5 eV corresponds to the carbon from O-C(O)-O of the carbonate group was approximately 1.6:1, which is in acceptable agreement with the expected ratio of 2:1.[3] Furthermore, the N 1s signal at 401.3 eV is intrinsic to the more electronegative nitrogen belong to the NHS group which showed a 2.3 eV shift in binding energy relative to the main N 1s peak at 398 eV which was similarly observed in other studies[4]. These results confirmed the activation of the hydroxyl group by DSC and hence the viability of the succinimidyl carbonate group to undergo a subsequent coupling step with an amine-containing entity.

The presence of adventitious carbonaceous contaminants on the surface also contributed to the measured peak intensity, which was also detected on bare SiN surface. Therefore, the ratio between the C 1s C-O and C 1s C-C peaks did not show a direct correlation to the number of EG monomer units present. Besides, the N 1s signal at 400 eV in Supplementary Fig. 6(f) is thought to be originated from impurities introduced during the DSC activation step.

On the other hand, it was not possible to deconvolute the O 1s envelope peak because of the presence of multiple organic species as well as possible signal contribution from adventitious oxygen on the surface and hence the O 1s signals were not analysed.

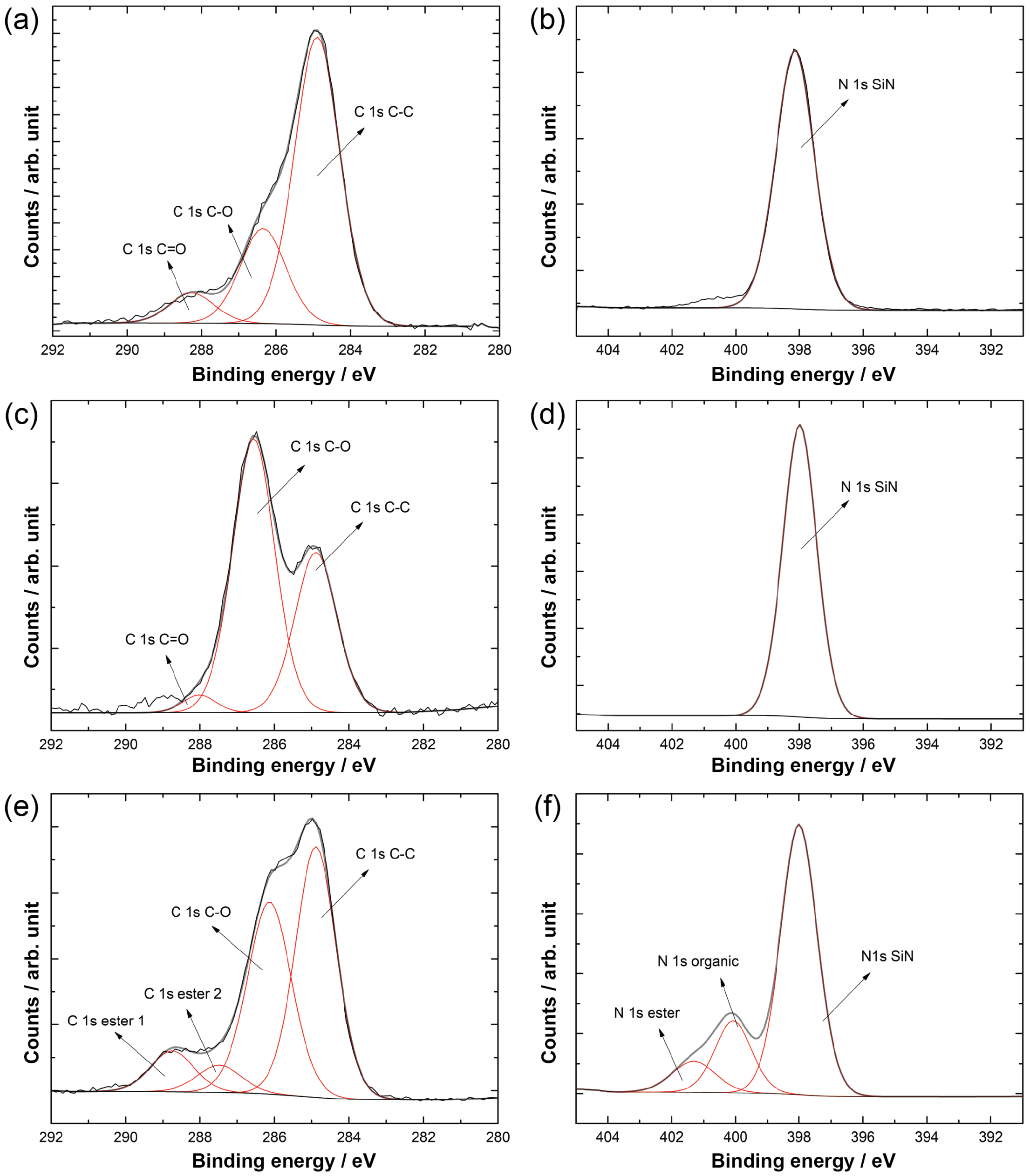

Supplementary Figure 6. X-ray photoelectron spectroscopy (XPS) characterisation of chemically modified SiN surfaces. (**a**) C 1s spectra and (**b**) N 1s spectra of bare SiN surface; (**c**) C 1s spectra and (**d**) N 1s spectra of silane-EG$_6$ SAM (OH-terminated) modified SiN surface; (**e**) C 1s spectra and (**f**) N 1s spectra of DSC-activated silane-EG$_6$ SAM modified SiN surface. Black and red solid lines denote fitted envelope signals and deconvoluted peaks respectively.

**1**	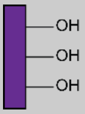<br>Bare SiN
**2**	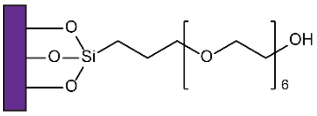<br>Silane-EG_6 (OH-terminated)
**3**	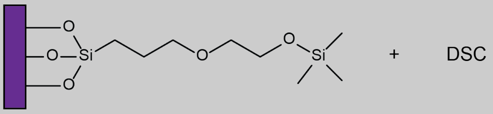<br>Silane-EG_6 (trimethylsilane protected) + DSC
**4**	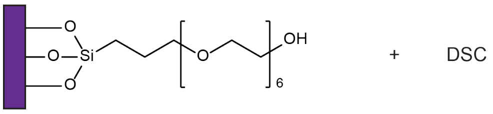<br>Silane-EG_6 (OH-terminated) + DSC

Supplementary Table 1. Prepared surfaces used in antifouling and DSC coupling studies.

To estimate the thickness of the surface chemistry layer inside the nanopores, a model that approximates the actual pore geometry and describes the ionic resistance (literally the inverse of ionic conductance, $G = 1/R$) of solid-state nanopores was formulated. For a truncated conical nanopore and assuming homogenous electrolyte conductivity (applicable for high ionic strength ≥100 mM KCl)[5], its ionic resistance can be expressed as[6]:

$$R_p = \frac{1}{\sigma}\int_{z=0}^{1}\frac{1}{A(z)}dx = \frac{4l}{\pi d_t d_b \sigma} \quad (2)$$

where σ is the bulk conductivity of electrolyte, z is the coordinate along the center axis of the pore, $A(z)$ is the cross-sectional area of the pore at position z, d_t and d_b are the diameters of the pore at the top and bottom of the SiN membrane, and l is the pore length which corresponds to thickness of the membrane layer.

As the electric field lines converge gradually from the bulk into the nanopore aperture, the variation of cross-sectional ion flux can have a significant effect on the overall pore resistance. This effect is known as access resistance, R_a, which can be approximated as the convergence of ionic resistance to a hemisphere of an equal radius as the pore[7]:

$$R_a = \frac{1}{2d\sigma} \tag{3}$$

Since there are two pore orifices in the SiN membrane and the resistance terms are additive, the total resistance of a truncated conical pore can be written as:

$$R = \frac{1}{\sigma}\left(\frac{4l}{\pi d_t d_b} + \frac{1}{2d_t} + \frac{1}{2d_b}\right) \tag{4}$$

For a nanopore with a diameter, d_b = 27 nm, the pore resistance was estimated to be 57.4 MΩ using the pore geometry as determined with SEM, which is in good agreement with the resistance value independently measured from the *I-V* curve (54.0 M MΩ) as shown in Supplementary Fig. 4(a). The bulk conductivity of the electrolyte, σ = 1.32 S m^{-1} for 100 mM KCl at 25°C was used to calculate the pore resistance[8]. As the ion flux traversing through the nanopores is extremely sensitive to changes in the nanopore geometry, it is possible to estimate the thickness of the SAMs inside nanopores by comparing the ionic resistance measured before and after the chemical modification step. It was found that the ionic resistance of a 27 nm nanopore in 100 mM KCl increased from 57 M MΩ to 74 M MΩ upon modification of the nanopore with a silane-EG_6 SAM as determined from the measured *I-V* curves as shown in Supplementary Fig. 7. This observation is consistent with a previously reported silane modified nanopore where the presence of a SAM on the internal pore wall resulted in a reduction of the effective pore diameter[9]. Using the nanopore ionic resistance model above, the increase of ionic resistance is commensurate with approximately 5.7 nm reduction of the effective pore diameter. In essence, this corresponds to an estimated monolayer thickness of 2.85 nm inside the nanopore.

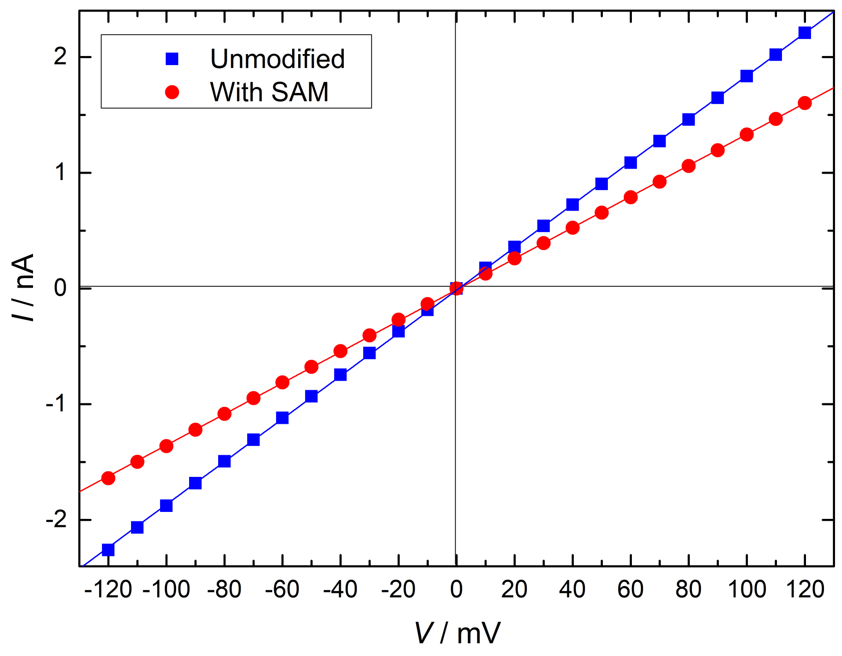

Supplementary Figure 7. Ionic current analysis of unmodified and silane-EG$_6$ modified solid-state nanopores. *I-V* curves of a 27 nm nanopore in SiN membrane before (blue) and after modification with silane-EG$_6$ SAM (red). Ionic currents were measured in 100 mM KCl and 10 mM Tris pH 7.4. Solid lines are linearly fitted to the ionic currents measured as a function of the applied potential bias.

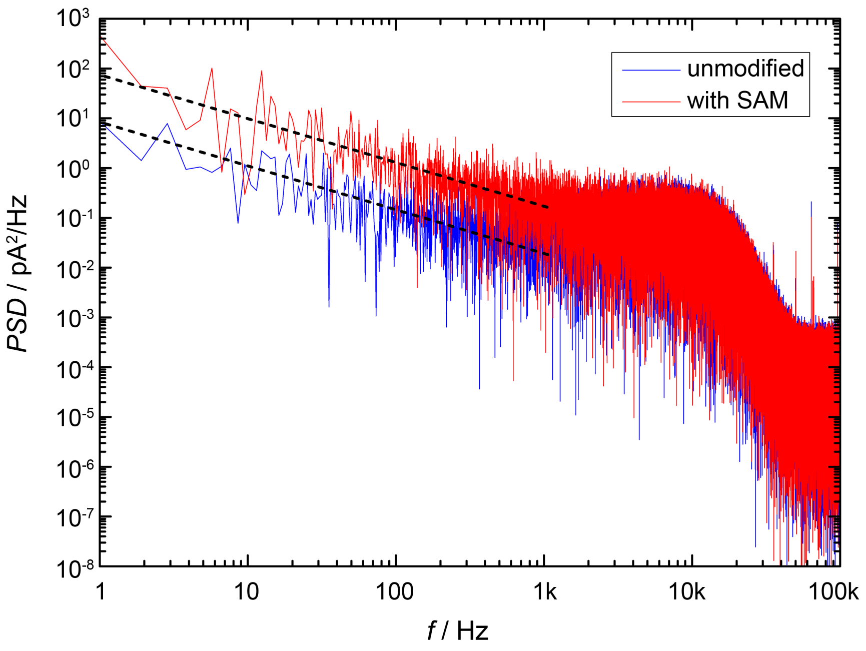

Supplementary Figure 8. Ionic current noise analysis of unmodified and silane-EG$_6$ SAM modified solid-state nanopores. Current noise PSD of a 30 nm nanopore before (blue)

and after chemical modification (red) with a silane-EG_6 monolayer where the potential difference was 100 mV. Black dashed lines are fitted to $S^2(f) \propto 1/f$.

It is important to ensure that the switching of the electromagnets as well as the magnetic field generated when the electromagnets were turned on do not impose any drastic effect on the ionic current measurements. Supplementary Figure 9 shows a short ionic current spike observed upon switching on the *cis*-magnet. Similar spikes were also detected when the *trans*-magnet was switched on as well as when the magnets were switched off afterwards. The resistive spikes observed after either *cis*- or *trans*-magnet was switched off were relatively longer in duration compared to the resistive spike caused by switching on the magnet. These transient changes in ionic current can be ascribed to the capacitive current generated in the patch clamp amplifier[10]. The spikes however, can be useful as tags to identify the switching events during analysis of the recorded ionic current traces. Nonetheless, the presence of the magnetic field did not change the ionic conductance as the ionic current maintained at a constant value before and after the electromagnets were switched on and then off. In addition, no additional ionic current noise was introduced when the electromagnets were switched on.

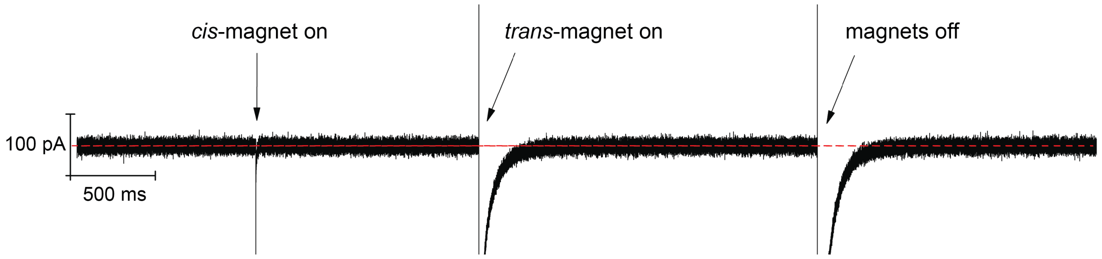

Supplementary Figure 9. Continuous ionic current trance of a 27 nm nanopore in 100 mM KCl and 10 mM Tris pH 7.4 when a potential bias of 100 mV is applied. The *cis*- and *trans*-magnets were each switched on and off individually as denoted during recording. No change in ionic conductance was observed after the electromagnets were switched on and off. The ionic current trace was recorded at 100 mV, low-pass-filtered at 10 kHz and sampled at 250 kHz. The red dashed line is shown as a guide to the eye.

Supplementary Note 5: Estimate of the number of anti-PSA antibody sites on a single MNP as well as a single nanopore

It is very challenging to unambiguously determine the number of immobilised anti-PSA antibodies on a single MNP as well as a single nanopore. Here the estimation was made by calculations based on simplified assumptions.

A simplified calculation was made with an assumption about the cross-section molecular size of 8.5 nm × 4.0 nm for an anti-PSA antibody and a 50% coupling efficiency for the attachment of anti-PSA antibodies onto it. Note that typical molecular dimensions for IgG protein are approximately 14.5 nm × 8.5 nm × 4.0 nm.[11] Also the molecular mass is around 150~170 KDa depending on a species. Here we calculate the possible number of attached anti-PSA antibodies on a MNP. Take a typical cubic MNP of 50 nm in length as an example, the total surface area for the cube would be 1.5×10^4 nm^2 (= 6 × 50 nm × 50 nm). So the possible maximum coverage (50%) for anti-PSA antibodies on it would be 7.5×10^3 nm^2. Therefore, the estimated number of anti-PSA antibodies would be about 220 (=7.5×10^3 nm^2/(8.5 nm × 4.0 nm)).

And so is the similar calculation for a functionalised nanopore. The interior surface area for a conical nanopore is about 15764 nm^2. So the estimated number for anti-PSA antibodies would be about 231. The nanopore interior surface area is calculated from the equation $\pi(r+R)S = \pi(r+R)\sqrt{[(R-r)^2+h^2]}$, where *r* and *R* stand for the radius of the bottom and top pore orifice (30 nm and 88 nm), respectively. And *h* stands for the membrane thickness (80 nm).

Supplementary Note 6: Characterisation of the magnetic nanoparticles

The synthesised MNPs were characterised under a scanning electron microscope (SEM) to determine the size and geometry of the nanoparticles. In Supplementary Fig. 10, the MNPs were found to be cubic and approximately 50 nm in size (average face-centred diagonal). The

cubic morphology of the MNPs was reported to be caused by the direct crystal growth mechanism of Fe_3O_4 during formation of MNPs in the presence of excess OH^-.[12]

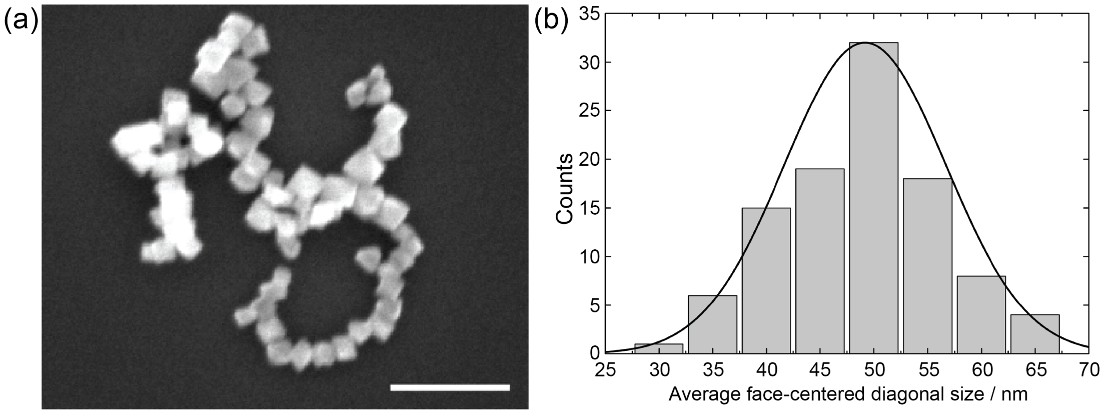

Supplementary Figure 10. Iron oxide magnetic nanoparticles. (**a**) Scanning electron micrograph showing the MNPs are cuboidal in shape. Scale bar: 200 nm. (**b**) The average size of the synthesised MNPs is 50 nm (face-centred diagonal). Black solid line is a Gaussian distribution function fit of the histogram.

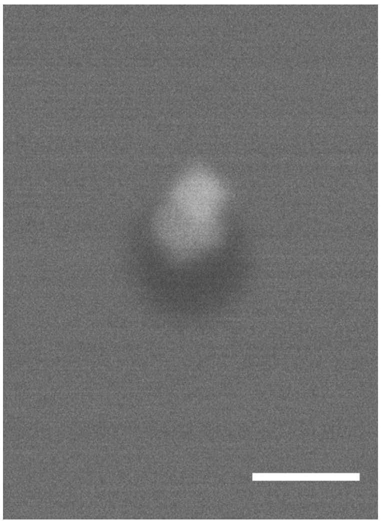

Supplementary Figure 11. SEM image of a MNP sitting inside a SiN nanopore. The image was took on a FEI Nova NanoSEM 450 instrument operated in backscattered electron imaging mode. Scale bar, 100 nm.

Supplementary Note 7: Finite Element Analysis

To understand the electrical potential profile inside a nanopore, COMSOL multiphysics finite element analysis (FEA) software (version 4.4) was used to compute the electric potential and

electric field distribution inside a nanopore. In the FEA simulations, a 30 nm nanopore was modelled to have a truncated cone geometry in a 180-nm-thick SiN. The SiN membrane was defined as an insulating region where no ionic current can pass through. In addition, the SiN membrane and nanopore surfaces were coated with a 3-nm-thick organic SAM layer with a relative permittivity, ε_r of 2.5[13]. Since the pore geometry is symmetric with respect to a rotation around the *z*-axis, the simulation model can be simplified into a 2D axial symmetric model as shown in Supplementary Fig. 12. In addition, the simulation did not include surface charge effects from the silane-EG_6 SAM to reduce computational requirements for the simulation since EG chains typically have a neutral charge density[14].

The continuous region outside of the insulating SiN membrane region represents the electrolyte medium. The thickness of the electrical double layer can be estimated by calculating the Debye length, r_D using the following expression:[15]

$$r_D = \sqrt{\frac{\varepsilon_r \varepsilon_0 k_B T}{2 N_A e^2 [\mathrm{KCl}]}} \tag{5}$$

where ε_0 is the permittivity of free space, ε_r is the dielectric constant of the electrolyte, k_B is the Boltzmann constant, T is the absolute temperature (298 K), N_A is Avogadro's number, e is the elementary charge and [KCl] is the concentration of the electrolyte used in mol/m^3. It was found that r_D = 0.95 nm for 100 mM KCl (ε_r = 77)[16]. This is much smaller than the dimensions of the nanopore and hence the electrolyte resistivity and concentration of the ionic species can be assumed to be homogenous across the entire electrolyte region. In the simulation model, the bulk ion diffusion coefficients of potassium, D_K and chloride, D_{Cl} at 298 K were assumed as 1.975×10^{-9} m^2 s^{-1} and 2.032×10^{-9} m^2 s^{-1} respectively[17].

A potential bias, V was applied across the insulating SiN membrane where the top and bottom boundaries of the simulated region were specified to have a potential of +100 mV and 0 mV respectively. A triangular-shaped adaptive mesh was used with a minimum element size of 0.5 nm and maximum element size of 169 nm. The mesh size is more refined in the vicinity

of the pore and at the interfaces as shown in Supplementary Fig. 12(a) to improve the accuracy of the simulations. Even finer mesh sizes did not yield any improvement to the solutions. Using the "Electrostatics Package" and "Transport of Diluted Species Package", the electric potential and field distributions in the simulated region were computed by solving the Nernst-Planck-Poisson equations. The simulated steady-state electric potential and field distributions are as illustrated in Supplementary Fig. 12(b) and quantitatively plotted in Supplementary Fig. 13.

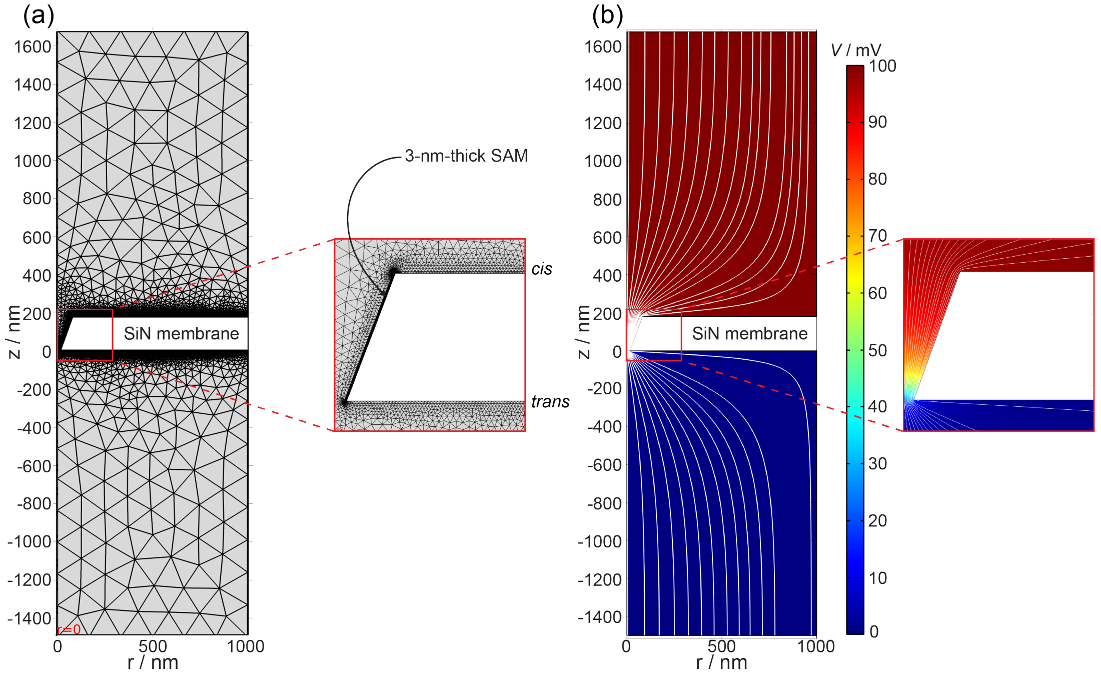

Supplementary Figure 12. Finite element analysis of electric potential and field distributions inside a nanopore. (**a**) An axial symmetric truncated conical nanopore coated with a 3-nm-thick SAM modelled in 2D with an adaptive mesh was used for the simulation. The mesh is much finer at the SAM interfacial region in vicinity of the nanopore (inset) to enhance simulation accuracy. (**b**) Electric potential distribution (colour map) and field lines (solid white lines) in the simulated region when a bias voltage of 100 mV is applied.

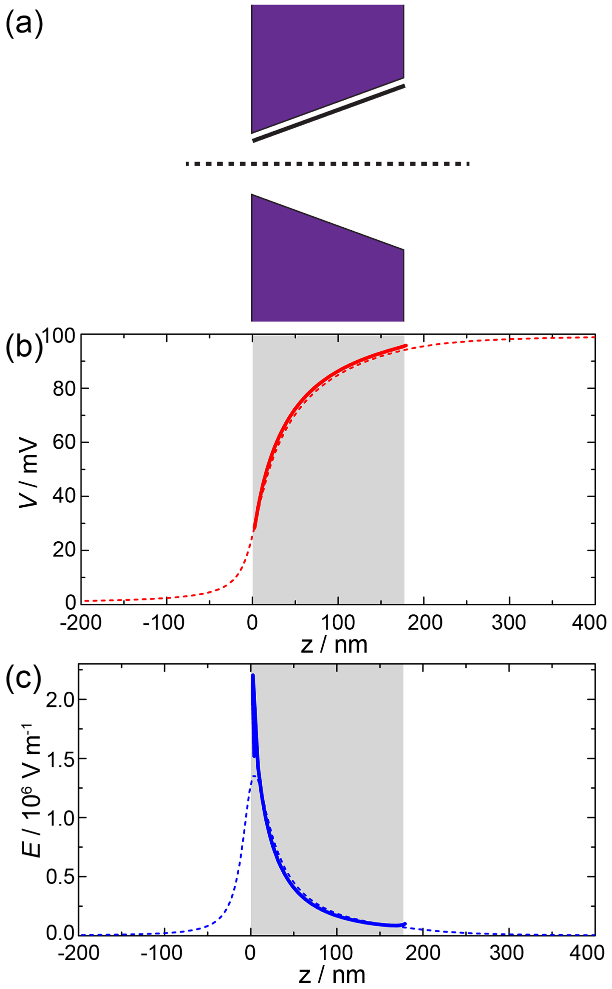

Supplementary Figure 13. Electric potential and field distributions inside a nanopore. (**b**) Distribution of electric potential and (**c**) magnitude of the electric field in a nanopore. Dashed lines denote the distributions along the center *z*-axis while solid lines represent distributions along a parallel-distance of 3 nm from the pore wall corresponding to the SAM interface as illustrated in (**a**).

Supplementary References

1 Wei, R., Pedone, D., Zuerner, A., Doeblinger, M. & Rant, U. Fabrication of metallized nanopores in silicon nitride membranes for single-molecule sensing. *Small* **6**, 1406-1414 (2010).

2 Gadaleta, A. *et al.* Sub-additive ionic transport across arrays of solid-state nanopores. *Phys. Fluids* **26**, 012005 (2014).

3 Boecking, T., Gal, M., Gaus, K. & Gooding, J. J. Evidence for why tri(ethylene oxide) functionalized Si-C linked monolayers on Si(111) have inferior protein antifouling properties relative to the equivalent alkanethiol monolayers assembled on gold. *Aust. J. Chem.* **58**, 660-663 (2005).

4 Bocking, T., James, M., Coster, H. G. L., Chilcott, T. C. & Barrow, K. D. Structural characterization of organic multilayers on silicon(111) formed by immobilization of molecular films on functionalized Si-C linked monolayers. *Langmuir* **20**, 9227-9235 (2004).

5 Smeets, R. M. *et al.* Salt dependence of ion transport and DNA translocation through solid-state nanopores. *Nano Lett.* **6**, 89-95 (2006).

6 Siwy, Z. & Fuliński, A. A nanodevice for rectification and pumping ions. *Am. J. Phys.* **72**, 567-574 (2004).

7 Hall, J. E. Access resistance of a small circular pore. *J. Gen. Physiol.* **66**, 531-532 (1975).

8 Vlassarev, D. M. & Golovchenko, J. A. Trapping DNA near a solid-state nanopore. *Biophys. J.* **103**, 352-356 (2012).

9 Mussi, V., Fanzio, P., Firpo, G., Repetto, L. & Valbusa, U. Size and functional tuning of solid state nanopores by chemical functionalization. *Nanotechnology* **23**, 435301 (2012).

10 Gold, R. *The Axon Guide - Electrophysiology and Biophysics Laboratory Techniques (3rd Edition)*, <https://www.moleculardevices.com/axon-guide> (2012).

11 Tan, Y. H., Liu, M., Nolting, B., Go, J. G., Gervay-Hague, J. & Liu, G. A nanoengineering approach for investigation and regulation of protein immbilization. *ACS Nano* **2**, 2374-2384 (2008).

12 Sugimoto, T. & Matijevic, E. Formation of uniform spherical magnetite particles by crystallization from ferrous hydroxide gels. *J. Colloid Interface Sci.* **74**, 227-243 (1980).

13 Polymeropoulos, E. E. & Sagiv, J. Electrical conduction through adsorbed monolayers. *J. Chem. Phys.* **69**, 1836-1847 (1978).

14 Revzin, A., Tompkins, R. G. & Toner, M. Surface engineering with poly(ethylene glycol) photolithography to create high-density cell arrays on glass. *Langmuir* **19**, 9855-9862 (2003).

15 Atkins, P. & de Paula, J. *Physical Chemistry*. (W. H. Freeman, New York, 2010).

16 Vanbeek, W. M. & Mandel, M. Static relative permittivity of some electrolyte solutions in water and methanol. *J. Chem. Soc. Faraday Trans. I* **74**, 2339-2351 (1978).

17 Suk, M. E. & Aluru, N. R. Ion transport in sub-5-nm graphene nanopores. *J. Chem. Phys.* **140**, 084707 (2014).
