# Supplementary material for: Nanopore blockade sensors for ultrasensitive detection of proteins in complex biological samples
Source: Nat Commun. 2019 May 8;10:2109. doi: 10.1038/s41467-019-10147-7 (PMC6506515; doi:10.1038/s41467-019-10147-7)
Supplement: Supplementary file 3 — Reporting Summary [file 41467_2019_10147_MOESM3_ESM.pdf]

nature research

Corresponding author(s): John Justin Gooding

Last updated by author(s): Mar 19, 2019

Reporting Summary

Nature Research wishes to improve the reproducibility of the work that we publish. This form provides structure for consistency and transparency in reporting. For further information on Nature Research policies, see Authors & Referees and the Editorial Policy Checklist.

Statistics

For all statistical analyses, confirm that the following items are present in the figure legend, table legend, main text, or Methods section.

n/a	Confirmed	
☐	☒	The exact sample size (n) for each experimental group/condition, given as a discrete number and unit of measurement
☐	☒	A statement on whether measurements were taken from distinct samples or whether the same sample was measured repeatedly
☐	☒	The statistical test(s) used AND whether they are one- or two-sided<br>*Only common tests should be described solely by name; describe more complex techniques in the Methods section.*
☒	☐	A description of all covariates tested
☒	☐	A description of any assumptions or corrections, such as tests of normality and adjustment for multiple comparisons
☐	☒	A full description of the statistical parameters including central tendency (e.g. means) or other basic estimates (e.g. regression coefficient) AND variation (e.g. standard deviation) or associated estimates of uncertainty (e.g. confidence intervals)
☒	☐	For null hypothesis testing, the test statistic (e.g. F, t, r) with confidence intervals, effect sizes, degrees of freedom and P value noted<br>*Give P values as exact values whenever suitable.*
☒	☐	For Bayesian analysis, information on the choice of priors and Markov chain Monte Carlo settings
☒	☐	For hierarchical and complex designs, identification of the appropriate level for tests and full reporting of outcomes
☒	☐	Estimates of effect sizes (e.g. Cohen's d, Pearson's r), indicating how they were calculated

Our web collection on statistics for biologists contains articles on many of the points above.

Software and code

Policy information about availability of computer code

Data collection	*Provide a description of all commercial, open source and custom code used to collect the data in this study, specifying the version used OR state that no software was used.*
Data analysis	*Provide a description of all commercial, open source and custom code used to analyse the data in this study, specifying the version used OR state that no software was used.*

For manuscripts utilizing custom algorithms or software that are central to the research but not yet described in published literature, software must be made available to editors/reviewers. We strongly encourage code deposition in a community repository (e.g. GitHub). See the Nature Research guidelines for submitting code & software for further information.

Data

Policy information about availability of data

All manuscripts must include a data availability statement. This statement should provide the following information, where applicable:

- Accession codes, unique identifiers, or web links for publicly available datasets
- A list of figures that have associated raw data
- A description of any restrictions on data availability

The authors declare that all data supporting the findings of this study are available within the paper and its Supplementary information and from the corresponding author upon reasonable request.

Field-specific reporting

Please select the one below that is the best fit for your research. If you are not sure, read the appropriate sections before making your selection.

☒ Life sciences ☐ Behavioural & social sciences ☐ Ecological, evolutionary & environmental sciences

For a reference copy of the document with all sections, see nature.com/documents/nr-reporting-summary-flat.pdf

Life sciences study design

All studies must disclose on these points even when the disclosure is negative.

Sample size	No sample size calculations were performed as the used statistical test works well for samples of small size. Description about sample size is documented in the relevant figure legend.
Data exclusions	No data exclusions were applied.
Replication	Number of repeats used to derive data is indicated in each figure legend. Typically this was at least 5 repeats.
Randomization	Randomization was not relevant to this study as this was a proof of concept study where the sensor performance was compared across samples including analyte molecules of different concentrations.
Blinding	It was not possible to do experiments with blinding as the chief experimentalist was in charge of sample preparation and sensing measurements.

Reporting for specific materials, systems and methods

We require information from authors about some types of materials, experimental systems and methods used in many studies. Here, indicate whether each material, system or method listed is relevant to your study. If you are not sure if a list item applies to your research, read the appropriate section before selecting a response.

Materials & experimental systems

n/a	Involved in the study	
☐	☒	Antibodies
☒	☐	Eukaryotic cell lines
☒	☐	Palaeontology
☒	☐	Animals and other organisms
☒	☐	Human research participants
☒	☐	Clinical data

Methods

n/a	Involved in the study	
☒	☐	ChIP-seq
☒	☐	Flow cytometry
☒	☐	MRI-based neuroimaging

Antibodies

Antibodies used	The two antibodies used in this study were purchased from Abcam company (UK). Anti-Prostate Specific Antigen antibodies used in this study were the mouse IgG monoclonal anti-PSA specific antibodies that one anti-PSA antibody (catalogue number ab10187, clone number 8A6) is specific for epitope 1 (only free PSA) while the other anti-PSA antibody (catalogue ab10185, clone number 5A6) is specific for epitope 5 of PSA (both free PSA and complexed PSA).
Validation	Abpromise guarantee covers the use of ab10187 in the following tested applications. The application notes include recommended starting dilutions; optimal dilutions/concentrations should be determined by the end user. Sandwich ELISA: Use at an assay dependent dilution. Best pair for detection of free PSA - (detection ab - capture ab) ab10187 - ab10185 For the detection of free PSA ab10187 can be used as a detection antibody in conjugation with ab10184. For the detection of free PSA ab10187 can be used as a detection antibody in conjugation with ab24466.<br>Abpromise guarantee covers the use of ab10185 in the following tested applications. Sandwich ELISA: Use at an assay dependent dilution. Can be paired for Sandwich ELISA with Mouse monoclonal [8A6] to Prostate Specific Antigen (ab10187). best pair for the detection of free PSA: (detection ab - capture ab) ab10187 - ab10185 For the detection of total PSA ab10185 can be used as capture antibody in conjunction with ab10184. For the detection of total PSA ab10185 can be used as a detection antibody in conjugation with ab10186.
